# (*Z*)-3-[(2-Fluoro­anilino)carbon­yl]prop-2-enoic acid

**DOI:** 10.1107/S1600536811001152

**Published:** 2011-01-15

**Authors:** Farooq Ali Shah, Saqib Ali, Saira Shahzadi, Sajjad Ahmad, Andreas Fischer

**Affiliations:** a Department of Chemistry, Quaid-i-Azam University, Islamabad 45320, Pakistan; b Department of Chemistry, GC University, Faisalabad, Pakistan; c Inorganic Chemistry, School of Chemical Science and Engineering, Royal Institute of Technology (KTH), 100 44 Stockholm, Sweden

## Abstract

In the title mol­ecule, C_10_H_8_FNO_3_, the dihedral angle between the fluoro­phenyl group and the essentially planar [within 0.064 (3) Å] COC=CCOOH unit, which has a *Z* configuration, is 19.99 (14)°. There is an intra­molecular O—H⋯O bond in the mol­ecule involving the acid –OH group and the adjacent carbonyl O atom. In the crystal, inter­molecular N—H⋯O bonds lead to the formation of polymer chains propagating along [011].

## Related literature

For the use of carb­oxy­lic acids containing N atoms as anti­biotics, see: Gould *et al.* (1980 ▶). For the biological properties of compounds containing keto, ester, imide and carboxylc acid groups, see: Chen & Njoroge (2009 ▶); Shen & Walford (1972 ▶; 1980 ▶). For the structure of 3-[(4-bromo­anilino)carbon­yl]prop-2-enoic acid, see Parvez, Shahid *et al.* (2004 ▶). For the structure of 3-[(2,4,6-tricholoroanilino)carbon­yl]prop-2-enoic acid, see Parvez, Shahzadi *et al.*(2004 ▶).
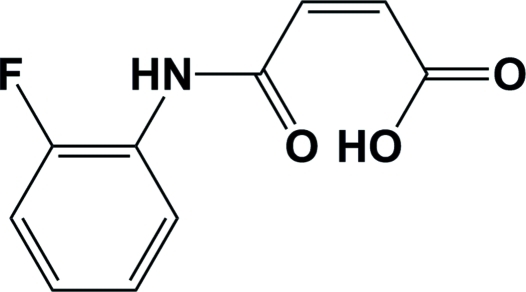


## Experimental

### 

#### Crystal data

C_10_H_8_FNO_3_
*M* *_r_* = 209.17
Orthorhombic, 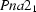

*a* = 20.282 (2) Å
*b* = 3.8025 (4) Å
*c* = 11.8183 (8) Å
*V* = 911.45 (15) Å^3^
*Z* = 4
Mo *K*α radiation
μ = 0.13 mm^−1^
*T* = 299 K
0.57 × 0.21 × 0.06 mm

#### Data collection

Bruker–Nonius KappaCCD diffractometer
7575 measured reflections
1075 independent reflections
799 reflections with *I* > 2σ(*I*)
*R*_int_ = 0.066

#### Refinement

*R*[*F* ^2^ > 2σ(*F* ^2^)] = 0.046
*wR*(*F* ^2^) = 0.121
*S* = 1.09
1075 reflections
132 parameters
1 restraint
H atoms treated by a mixture of independent and constrained refinement
Δρ_max_ = 0.27 e Å^−3^
Δρ_min_ = −0.27 e Å^−3^

### 

Data collection: *COLLECT* (Nonius, 1998 ▶); cell refinement: *DIRAX* (Duisenberg, 1992 ▶); data reduction: *EVALCCD* (Duisenberg *et al.*, 2003 ▶); program(s) used to solve structure: *SHELXS97* (Sheldrick, 2008 ▶); program(s) used to refine structure: *SHELXL97* (Sheldrick, 2008 ▶); molecular graphics: *DIAMOND* (Brandenburg, 2007 ▶); software used to prepare material for publication: *SHELXL97* and *publCIF* (Westrip, 2010 ▶).

## Supplementary Material

Crystal structure: contains datablocks I, global. DOI: 10.1107/S1600536811001152/su2241sup1.cif

Structure factors: contains datablocks I. DOI: 10.1107/S1600536811001152/su2241Isup2.hkl

Additional supplementary materials:  crystallographic information; 3D view; checkCIF report

## Figures and Tables

**Table 1:** Hydrogen-bond geometry (Å, °)

*D*—H⋯*A*	*D*—H	H⋯*A*	*D*⋯*A*	*D*—H⋯*A*
O3—H3*B*⋯O1	0.92 (8)	1.60 (8)	2.506 (4)	170 (7)
N1—H1*A*⋯O2^i^	0.83 (5)	2.10 (5)	2.929 (5)	175 (4)

Symmetry code: (i) 
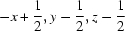
.

**Fig. 1.**
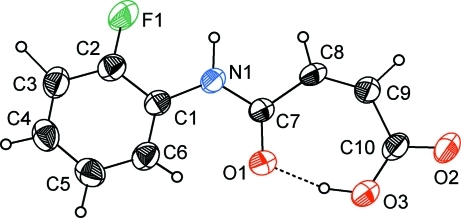
The molecular structure of the title compound. Thermal ellipsoids are drawn at the 50% probability level. The intramolecular O—H···O hydrogen bond is shown as a dashed line.

**Fig. 2.**
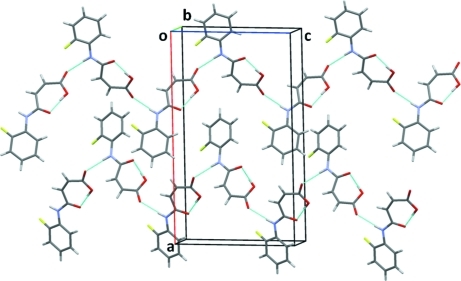
A view along the b axis of the crystal packing of the title compound. The hydrogen bonds are shown as dashed cyan lines (see Table 1 for details).

## supplementary crystallographic information

### Comment

Carboxylic acids are good reducing agents and applied as an antioxidant agent and a precursor for the prevention of cancer. Carboxylic acids are used for therapeutic purposes and for other biological applications as well. Those carboxylic acids which contain N atoms are widely used as antibiotics (Gould *et al.*, 1980). Compounds containing NH and carboxyl groups are of great interest for the synthesis of drugs due to their coordination to biological systems. Compounds containing keto, ester and imide are good anti-HCV agents (Chen *et al.*, 2009). Carboxylic acids are also used as anti-inflammatories (Shen & Walford, 1980) and enzymetic inhibitors (Shen & Walford, 1972). We therefore used maleic anhydride to synthesize the title compound, by condensation with 2-fluoroaniline.

The molecular structure of the title compound is illustrated in Fig. 1. The bond distances and angles are close to those observed in simlar compounds; 3-[(4-bromoanilino)carbonyl]prop-2-enoic acid (Parvez, Shahid *et al.*, 2004) and 3-[(2,4,6-Tricholoroanilino)carbonyl]prop-2-enoic acid (Parvez, Shahzadi *et al.*, 2004). In the molecule there is an intramolecular O—H···O hydrogen bond involving the acid OH group and the adjacent carbonyl O-atom (Table 1). The COC=CCOOH moiety is essentially planar [atoms O1,C7—C10, O2,O3 planar to within 0.064 (3) Å], and has a *Z* configuration. It makes a dihedral angle of 19.99 (14) Å with the phenyl ring.

In the crystal there are intermolecular N–H···.O hydrogen bonds connecting symmetry related molecules yielding chains propagating along [011] (Table 1 and Fig. 2).

### Experimental

A solution of maleic anhydride (5 g, 1 mmol) in (300 ml glacial acetic acid) was added to a solution of 2-fluoroaniline (5 ml, 1 mmol) (150 ml glacial acetic acid) in 500 ml beaker at room temperature and the mixture was stirred in fuming hood at room temperature overnight. The light yellow precipitates formed were filtered off, washed with cold distilled H_2_O (200 ml) and air dried. The yellow crystals, suitable for X-ray diffraction analysis, were obtained by recrystallization in acetone:n-hexane (1:1).

### Refinement

In the final cycles of refinement, in the absence of significant anomalous scattering effects, 719 Friedel pairs were merged and Δf " set to zero. The OH and NH H-atoms were located from a difference Fourier map and were freely refined: O3—H3B = 0.92 (8) Å, N1—H1A = 0.83 (5) Å. The C-bound H-atoms were included in calculated positions and treated as riding atoms: C—H = 0.93 Å with *U*_iso_(H) = 1.2*U*_eq_(parent C-atom).

### Crystal data

**Table:**

C_10_H_8_FNO_3_	*F*(000) = 432
*M**_r_* = 209.17	*D*_x_ = 1.524 Mg m^−^^3^
Orthorhombic, *P**n**a*2_1_	Mo *K*α radiation, λ = 0.71073 Å
Hall symbol: P 2c -2n	Cell parameters from 49 reflections
*a* = 20.282 (2) Å	θ = 6.2–19.7°
*b* = 3.8025 (4) Å	µ = 0.13 mm^−^^1^
*c* = 11.8183 (8) Å	*T* = 299 K
*V* = 911.45 (15) Å^3^	Plate, yellow
*Z* = 4	0.57 × 0.21 × 0.06 mm

### Data collection

**Table:**

Bruker–Nonius KappaCCD diffractometer	799 reflections with *I* > 2σ(*I*)
Radiation source: fine-focus sealed tube	*R*_int_ = 0.066
graphite	θ_max_ = 27.5°, θ_min_ = 5.3°
φ and ω scans	*h* = −24→26
7575 measured reflections	*k* = −4→4
1075 independent reflections	*l* = −15→15

### Refinement

**Table:**

Refinement on *F*^2^	Primary atom site location: structure-invariant direct methods
Least-squares matrix: full	Secondary atom site location: difference Fourier map
*R*[*F*^2^ > 2σ(*F*^2^)] = 0.046	Hydrogen site location: inferred from neighbouring sites
*wR*(*F*^2^) = 0.121	H atoms treated by a mixture of independent and constrained refinement
*S* = 1.09	*w* = 1/[σ^2^(*F*_o_^2^) + (0.0562*P*)^2^ + 0.3218*P*] where *P* = (*F*_o_^2^ + 2*F*_c_^2^)/3
1075 reflections	(Δ/σ)_max_ < 0.001
132 parameters	Δρ_max_ = 0.27 e Å^−^^3^
1 restraint	Δρ_min_ = −0.27 e Å^−^^3^

### Special details

**Table:**

Geometry. Bond distances, angles *etc*. have been calculated using the rounded fractional coordinates. All su's are estimated from the variances of the (full) variance-covariance matrix. The cell e.s.d.'s are taken into account in the estimation of distances, angles and torsion angles
Refinement. Refinement of *F*^2^ against ALL reflections. The weighted *R*-factor *wR* and goodness of fit *S* are based on *F*^2^, conventional *R*-factors *R* are based on *F*, with *F* set to zero for negative *F*^2^. The threshold expression of *F*^2^ > σ(*F*^2^) is used only for calculating *R*-factors(gt) *etc*. and is not relevant to the choice of reflections for refinement. *R*-factors based on *F*^2^ are statistically about twice as large as those based on *F*, and *R*- factors based on ALL data will be even larger.

### Fractional atomic coordinates and isotropic or equivalent isotropic displacement parameters (Å^2^)

**Table:**

	*x*	*y*	*z*	*U*_iso_*/*U*_eq_	
F1	0.06487 (13)	0.1290 (8)	0.1972 (2)	0.0682 (9)	
O1	0.13208 (13)	0.4940 (9)	0.5666 (2)	0.0568 (9)	
O2	0.31277 (15)	1.0159 (9)	0.6674 (3)	0.0608 (10)	
O3	0.21896 (15)	0.7409 (10)	0.6947 (3)	0.0609 (12)	
N1	0.11309 (15)	0.4479 (9)	0.3789 (3)	0.0380 (10)	
C1	0.04662 (8)	0.3316 (7)	0.38017 (19)	0.0339 (10)	
C2	0.02363 (10)	0.1799 (7)	0.28070 (18)	0.0440 (12)	
C3	−0.04193 (11)	0.0770 (7)	0.2720 (2)	0.0510 (16)	
C4	−0.08451 (9)	0.1257 (8)	0.3629 (2)	0.0500 (15)	
C5	−0.06152 (10)	0.2773 (8)	0.4623 (2)	0.0491 (15)	
C6	0.00405 (11)	0.3803 (7)	0.47100 (17)	0.0436 (11)	
C7	0.15083 (18)	0.5352 (10)	0.4677 (3)	0.0376 (11)	
C8	0.21585 (19)	0.6799 (11)	0.4379 (3)	0.0418 (11)	
C9	0.26209 (19)	0.8086 (11)	0.5039 (3)	0.0426 (11)	
C10	0.2656 (2)	0.8609 (12)	0.6285 (3)	0.0433 (14)	
H1A	0.132 (2)	0.467 (11)	0.317 (4)	0.040 (12)*	
H3	−0.05730	−0.02450	0.20550	0.0610*	
H3B	0.190 (4)	0.630 (18)	0.647 (7)	0.11 (2)*	
H4	−0.12840	0.05680	0.35710	0.0600*	
H5	−0.09000	0.30990	0.52310	0.0590*	
H6	0.01940	0.48170	0.53760	0.0520*	
H8	0.22570	0.68040	0.36100	0.0500*	
H9	0.29970	0.88070	0.46550	0.0510*	

### Atomic displacement parameters (Å^2^)

**Table:**

	*U*^11^	*U*^22^	*U*^33^	*U*^12^	*U*^13^	*U*^23^
F1	0.0578 (14)	0.107 (2)	0.0399 (13)	−0.0096 (15)	0.0025 (12)	−0.0258 (14)
O1	0.0460 (14)	0.096 (2)	0.0283 (14)	−0.0165 (15)	−0.0017 (13)	0.0017 (16)
O2	0.0468 (16)	0.093 (2)	0.0425 (17)	−0.0111 (16)	−0.0097 (14)	−0.0176 (17)
O3	0.0479 (16)	0.101 (3)	0.0337 (15)	−0.0106 (18)	−0.0026 (14)	−0.0121 (19)
N1	0.0357 (15)	0.050 (2)	0.0284 (16)	0.0003 (14)	−0.0007 (15)	−0.0046 (16)
C1	0.0368 (17)	0.0353 (18)	0.0297 (19)	0.0044 (15)	−0.0042 (16)	0.0032 (16)
C2	0.044 (2)	0.046 (2)	0.042 (2)	0.0008 (18)	−0.003 (2)	0.001 (2)
C3	0.052 (2)	0.049 (3)	0.052 (3)	−0.0084 (19)	−0.017 (2)	0.003 (2)
C4	0.0400 (19)	0.054 (3)	0.056 (3)	−0.0057 (18)	−0.007 (2)	0.017 (2)
C5	0.0363 (18)	0.062 (3)	0.049 (3)	0.0049 (18)	0.0005 (19)	0.015 (2)
C6	0.0417 (19)	0.049 (2)	0.040 (2)	0.0044 (18)	−0.0049 (18)	0.001 (2)
C7	0.0366 (19)	0.046 (2)	0.0302 (19)	0.0001 (16)	0.0000 (17)	−0.0015 (19)
C8	0.043 (2)	0.058 (2)	0.0244 (17)	−0.0028 (19)	0.0019 (16)	−0.0086 (17)
C9	0.0339 (18)	0.059 (2)	0.035 (2)	−0.0034 (18)	0.0026 (16)	−0.0062 (19)
C10	0.037 (2)	0.059 (3)	0.034 (2)	0.0081 (19)	−0.0041 (18)	−0.008 (2)

### Geometric parameters (Å, °)

**Table:**

F1—C2	1.308 (3)	C4—C5	1.3892
O1—C7	1.239 (4)	C5—C6	1.3902
O2—C10	1.214 (5)	C7—C8	1.472 (5)
O3—C10	1.310 (5)	C8—C9	1.314 (5)
O3—H3B	0.92 (8)	C9—C10	1.488 (5)
N1—C1	1.419 (4)	C3—H3	0.9300
N1—C7	1.341 (5)	C4—H4	0.9300
N1—H1A	0.83 (5)	C5—H5	0.9300
C1—C6	1.3900	C6—H6	0.9300
C1—C2	1.3900	C8—H8	0.9300
C2—C3	1.3899	C9—H9	0.9300
C3—C4	1.3908		
			
C10—O3—H3B	105 (5)	C7—C8—C9	129.5 (3)
C1—N1—C7	127.7 (3)	C8—C9—C10	132.2 (4)
C1—N1—H1A	118 (3)	O2—C10—O3	120.8 (4)
C7—N1—H1A	114 (3)	O2—C10—C9	118.5 (4)
N1—C1—C2	116.1 (2)	O3—C10—C9	120.7 (4)
N1—C1—C6	123.8 (2)	C2—C3—H3	120.00
C2—C1—C6	120.00	C4—C3—H3	120.00
C1—C2—C3	120.02	C3—C4—H4	120.00
F1—C2—C1	119.0 (2)	C5—C4—H4	120.00
F1—C2—C3	121.0 (2)	C4—C5—H5	120.00
C2—C3—C4	119.96	C6—C5—H5	120.00
C3—C4—C5	120.00	C1—C6—H6	120.00
C4—C5—C6	120.04	C5—C6—H6	120.00
C1—C6—C5	119.97	C7—C8—H8	115.00
O1—C7—N1	122.1 (3)	C9—C8—H8	115.00
O1—C7—C8	123.2 (3)	C8—C9—H9	114.00
N1—C7—C8	114.6 (3)	C10—C9—H9	114.00
			
C7—N1—C1—C2	−165.3 (3)	F1—C2—C3—C4	178.4 (3)
C7—N1—C1—C6	18.5 (5)	C1—C2—C3—C4	−0.04
C1—N1—C7—O1	6.3 (6)	C2—C3—C4—C5	0.04
C1—N1—C7—C8	−174.2 (3)	C3—C4—C5—C6	−0.02
N1—C1—C2—F1	5.3 (4)	C4—C5—C6—C1	0.00
N1—C1—C2—C3	−176.3 (3)	O1—C7—C8—C9	−5.0 (7)
C6—C1—C2—F1	−178.4 (3)	N1—C7—C8—C9	175.4 (4)
C6—C1—C2—C3	0.03	C7—C8—C9—C10	−1.3 (8)
N1—C1—C6—C5	176.0 (3)	C8—C9—C10—O2	−173.7 (5)
C2—C1—C6—C5	−0.02	C8—C9—C10—O3	6.6 (7)

### Hydrogen-bond geometry (Å, °)

**Table:**

*D*—H···*A*	*D*—H	H···*A*	*D*···*A*	*D*—H···*A*
O3—H3B···O1	0.92 (8)	1.60 (8)	2.506 (4)	170 (7)
N1—H1A···O2^i^	0.83 (5)	2.10 (5)	2.929 (5)	175 (4)

Symmetry codes: (i) −*x*+1/2, *y*−1/2, *z*−1/2.
